# Studies of pathology and pharmacology of diabetic encephalopathy with KK‐Ay mouse model

**DOI:** 10.1111/cns.13201

**Published:** 2019-08-11

**Authors:** Si Shi, Hua‐Jing Yin, Jiang Li, Ling Wang, Wei‐Ping Wang, Xiao‐Liang Wang

**Affiliations:** ^1^ Department of Pharmacology, State Key Laboratory of Bioactive Substances and Functions of Natural Medicines, Institute of Materia Medica Chinese Academy of Medical Sciences and Peking Union Medical College Beijing China

**Keywords:** diabetic encephalopathy, GFAP, KK‐Ay, MAP2, PHPB

## Abstract

**Aims:**

Pathogenesis of diabetic encephalopathy (DE) is not completely understood until now. The purposes of this study were to illustrate the changes in morphology, function, and important transporters in neurons and glia during DE, as well as to reveal the potential therapeutic effects of medicines and the diet control on DE.

**Methods:**

Spontaneous obese KK‐Ay mice were used to investigate diabetes‐induced cognitive disorder, the morphology, function, and protein expression changes in impact animal and the cell level studies. The new drug candidate PHPB, donepezil, and low‐fat food were used to observe the therapeutic effects.

**Results:**

KK‐Ay mice at 5 months of age showed typical characteristics of type 2 diabetes mellitus (T2DM） and appeared significant cognitive deficits. Morphological study showed microtubule‐associated protein 2 (MAP2) expression was increased in hippocampal neurons and glial fibrillary acidic protein (GFAP) expression decreased in astrocytes. Meanwhile, the vesicular glutamate transporter 1 (vGLUT1) expression was increased and glucose transporter 1 (GLUT1) decreased, and the expression of brain‐derived neurotrophic factor (BDNF) and glial cell‐derived neurotrophic factor (GDNF) was also reduced in KK‐Ay mice. Microglia were activated, and IL‐1β and TNF‐α were increased obviously in the brains of the KK‐Ay mice. Most of the above changes in the KK‐Ay mice at 5 months of age could be relieved by diet intervention (DR) or by treatment of donepezil or new drug candidate PHPB.

**Conclusion:**

KK‐Ay mouse is a useful animal model for studying DE. The alterations of morphology, structure, and function of astrocyte and microglia in KK‐Ay mice might be rescued by DR and by treatment of medicine. The proteins we reported in this study could be used as biomarkers and the potential drug targets for DE study and treatment.

## 1 INTRODUCTION

It has been demonstrated that type 2 diabetes mellitus (T2DM）is a major risk factor of the dementia.^1^, ^2^ Diabetes‐related cognitive dysfunction with the altered morphology and abnormal neuronal physiological function has been defined as diabetic encephalopathy (DE).^3^, ^4^ Most of the earlier studies focused on the similarity between DE and vascular dementia (VD), but researchers have recently found that DE is also associated with Alzheimer's disease (AD).^5^ Epidemiological data showed that patients of T2DM are about two times higher risk for dementia compared to the people without diabetes, and currently, one in 10 cases of dementia can be attributed to T2DM.^5^ Because of the increased incidence of T2DM in worldwide, DE has become a serious medical and social issue. Therefore, it is important to reveal the pathological mechanisms in DE and find therapeutic strategies.

Hippocampus, the key area for learning and memory in the brain, is particularly sensitive to changes in glucose and lipid homeostasis.^6^, ^7^ Recently, both astrocyte and microglia, which is not only taking charge in supporting survival and function of neuron but also affecting the capability of learning and memory directly,^8^, ^9^ have been caused more and more attention in the DE researches. A great body of evidence has shown that blood‐brain barrier (BBB) injury, energy metabolic disorder, electrophysiological deficits, neuroinflammation, neurotransmitter change, and neuronal apoptosis were found both in the brain of T2DM patients and diabetic animal models and could be involved in the pathological mechanism of DE.^1^, ^6^, ^10^, ^11^, ^12^, ^13^ However, different studies had yielded conflicting results. For example, some studies had indicated that severe loss of cerebral neuron was observed in the diabetic rat in the early stage, but other reports had shown that there was just moderate degeneration of cerebral neuron along in the rat with the diabetes development. 1, 2, 6, 7 Moreover, several researches had found astrogliosis, glutamate excitotoxicity occurred in the brain of diabetic animal models, when other reports showed no obvious change in them or even had opposite results.^1^, ^11^ It might be attributed to the differences in experimental models and the observation time. The streptozotocin (STZ)‐induced diabetic rat was used in most studies; however, it is difficult to mimic T2DM of humans which is a heterogeneous and multifactorial disease.^14^ The KK‐Ay mouse, transferred the yellow obese gene (Ay allele) into the Kuo Kondo (KK) mouse which spontaneously developed to severe obesity, hyperglycemia, and insulin resistance, was established as a suitable and useful model mimicking human obesity and T2DM.^15^ So far, the study of obese diabetes inducing cognitive deficits was scarcely reported in KK‐Ay mice. In our previous study, we had found a high incidence of dementia in KK‐Ay mice.^16^

In the present study, we simultaneously observed the histological and functional change in both neuron and glia in the hippocampus of 3‐, 5‐, and 7‐month‐old KK‐Ay mice to elucidate the mechanism of DE in the duration of the disease. In addition, we investigated the protective effects of two agents on DE, one is donepezil which is a classical agent for AD and another is *dl*‐PHPB (potassium 2‐(1‐hydroxypentyl)‐benzoate) which is a novel drug candidate for the treatment of cerebral ischemic stroke. PHPB is the prodrug of listed antiischemia agent 3‐n‐butylphthalide (dl‐NBP),^17^ and recently, it was reported to be potent for AD^18^ and VD in animal models.^19^ *Dl*‐PHPB is now in phase II clinical studies for ischemic stroke. Our present study is helpful to understand the difference between DE and AD and can provide some valuable information for early diagnose and treatment of DE.

## 2 MATERIALS AND METHODS

### 2.1 Animal and treatments

Male KK/Upj‐Ay/J mice (Beijing HFK Bioscience Co. Ltd.) were fed with a high‐fat diet until the experiments were carried out at 3, 5, and 7 months of age. Age‐matched male C57BL/6J mice (WT mice; Beijing Vital River Laboratory Animal Technology Co, Ltd) were generally employed as nondiabetic control for the KK‐Ay mice.

Male KK‐Ay mice were randomly divided into four groups: untreated group (KK‐Ay, given high‐fat diet) and treated groups. Treated groups were received *dl*‐PHPB (PHPB; Yunnan Haobang Pharmaceutical Co. Ltd, 150 mg/kg/day) or donepezil group (Don, Eisai Co, Ltd., 3 mg/kg/day) by oral gavage, or diet intervention (DR) group by giving a normal low‐fat diet. All treatment continued for two months beginning at 3 months of age. Age‐matched C57BL/6J mice were used as the control group (WT) by treatment with physiological saline.

Mice were group‐housed in an animal room at a constant temperature of 23 ± 1°C and maintained at a 12 hours light/dark cycle per day. All animals were given food and water ad libitum. All experiments were approved and performed in accordance with the institutional guidelines of the Experimental Animal Center of the Chinese Academy of Medical Science, Beijing, China.

### 2.2 Morris water maze

The spatial learning and memory of rats were tested according to the method of R. Morris.^9^ Briefly, the mouse was gently released into a circle metal pool (120 cm in diameter), filled with water made opaque by the addition of milk powder, from one of the four preplanned starting positions. Spatial training of the hidden platform (submerged 1 cm below the water) in the water maze was performed for five consecutive days. On each day, the mice were allowed to consecutive training trials of which allowed the mouse to swim for a maximum duration of 120 seconds in each trial to find the hidden platform. The duration for finding the platform was recorded as latency. The mice both of which found the hidden platform or not allowed to stay on the platform for 30 seconds. The mouse with blindness was excluded.

### 2.3 Biochemistry index

Blood samples were collected by cutting the tail or from the heart of mice anesthetized with 4% chloral hydrate in the end of the experimental period. Random plasma glucose, cholesterol, and triglyceride were measured by using test kits (Nanjing Jiancheng Bioengineering Institute, China), and random plasma insulin was analyzed using enzyme‐linked immunosorbent assay (ELISA) (DRG Diagnostics).

### 2.4 Histological staining

After perfusion through the heart in the deeply anesthetized mice, the brains were taken out. The right hemisphere was used for hematoxylin‐eosin (HE) and immunofluorescence staining. The hippocampus was dissected from the left hemispheres for western blotting (WB) and ELISA. The right hemisphere was immediately immersed into 4% paraformaldehyde for 18 hours; then, it was sequentially transformed into 10%, 20%, 30% sucrose solution, with 24 hours each time. After complete dehydration, the tissue was frozen and then prepared for frozen section. The brain was cut serially on a Leica microtome into a 20‐μm‐thick coronal section. After immersing in PBS for 30 minuts to get rid of the OCT, all coronal sections were mounted on glass slides and stained with the routine HE technique.^20^

### 2.5 Immunofluorescence staining

The coronal sections were incubated with 10% normal donkey serum to block nonspecific binding, followed by an overnight incubation with the primary antibodies, anti‐NeuN (1:500, Millipore), GFAP (1:400, Dako, DK), S100B (1:500, Abcam), IBA‐1 (Wako Pure Chemical Industries, Ltd) at 4°C. After washing, the sections were incubated with secondary antibodies, including Alexa Fluor 488‐conjugated donkey antimouse IgG (1:200; Thermo Fisher Scientific) or Alexa Fluor 594‐conjugated donkey antirat IgG (1:200, Thermo Fisher Scientific) for 2 hours at room temperature. The sections were rinsed and transferred on slides, and the cover slipped in an antifade agent. The images were acquired using a Nikon camera mounted on a Nikon Eclipse 80i microscope (Nikon) and analyzed using Image J software. The threshold of detection was held constantly during analysis.

### 2.6 Western blotting analysis

Equal amounts of protein per lane were loaded and performed electrophoresis and then transferred to polyvinylidene fluoride membranes. The membrane was blocked with 5% milk for 2 hours and then incubated with the following primary antibodies overnight at 4°C, anti‐NeuN (1:1000, Millipore), MAP2 (1:500, Sigma Aldrich), GFAP (1:1000; Cell Signaling Technology), GLUT1 (1:500, Abcam), vGLUT1 (1:400, Abcam), EAAT2 (1:400, Santa), and β‐actin (1:10 000, Cell Signaling Technology). The membranes were rinsed in Tris‐buffered saline with 0.1% Tween‐20, incubated with horseradish peroxidase‐conjugated secondary antibodies at room temperature for 1 hour, and detected using an enhanced chemiluminescence (ECL) kit. Densitometric evaluation was analyzed using the Quantity One image analysis software (Bio‐Rad). β‐Actin was used as a loading control.

### 2.7 Enzyme‐Linked Immunosorbent Assay (ELISA)

BDNF, GDNF, IL‐1β, and TNF‐α in the hippocampal homogenate were detected by ELISA according to the manufacture's instruction (Cusabio). The values were referred as the relative amount per total protein (pg/mg).

### 2.8 Statistical analysis

All data were expressed as mean ± SEM. Repeated measures analysis of variance or Student's *t* test or two‐way analysis of variance (ANOVA) followed by post hoc LSD test was used for multiple comparisons (SPSS version 19.0, SPSS Inc). Statistical significance was set to a value of *P* *<* .05.

## 3 RESULT

### 3.1 The effects of PHPB, donepezil, and diet intervention on the metabolic syndrome and spatial learning and memory deficits in KK‐Ay mice

**Figure 1:**
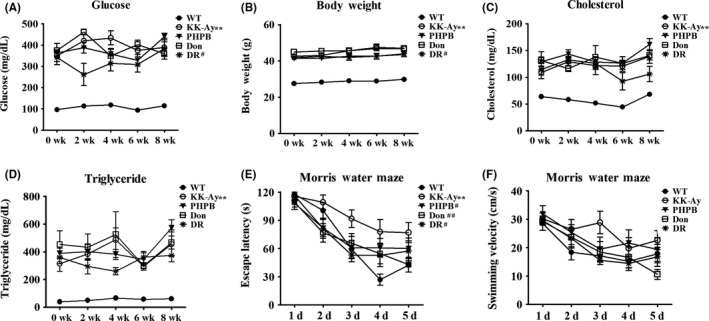
The changes of biochemical indexes and cognitive behaviors in 5‐mo‐old KK‐Ay mice and the improvements by PHPB, donepezil, and diet intervention (DR). A‐D, Random blood glucose, body weight, cholesterol, and triglyceride were significantly enhanced in KK‐Ay mice. No obvious changes in glucose, cholesterol, and triglyceride were detected in KK‐Ay mice after treatment with PHPB 150 mg/kg or donepezil (Don) 3 mg/kg for two months, but DR for two months decreased the glucose and body weight in KK‐Ay mice. E, The escape latency of 5‐mo‐old KK‐Ay mice to find the hidden platform was longer than WT mice in Morris Water Maze. All KK‐Ay mice in PHPB, donepezil, and DR groups showed good performance. F, No obvious difference in swimming velocity was observed in both KK‐Ay and WT mice of all groups, n = 12‐16 per group. ^**^ *P* < .01 versus WT mice, ^#^ *P* *<* .05, ^##^ *P* *<* .01 versus KK‐Ay mice

Our previous study had shown that the body weight, random blood glucose, cholesterol, triglyceride, and insulin of KK‐Ay mice were significantly increased compared to control age‐matched group at 3,5, and 7 months of age, which suggested that KK‐Ay is a reliable model of T2DM.^16^ Furthermore, KK‐Ay mice showed obvious spatial learning and memory deficits in the diabetic duration.^16^ In the present study, we found that PHPB (150 mg/kg) and donepezil (3 mg/kg) treatment had no significant effect on the biochemistry index of 5‐month‐old KK‐Ay mice but DR could reduce the blood glucose and body weight (Figure 1A‐D). In Morris water maze, all treatment groups showed improvements in behavioral performance without any obvious differences in swimming velocity (Figure 1E,F). These results optimistically indicated that the cognitive deficits in KK‐Ay mice were reversible and could be prevented at an early stage.

### 3.2 Changes in neurons in the hippocampus of KK‐Ay mice

**Figure 2:**
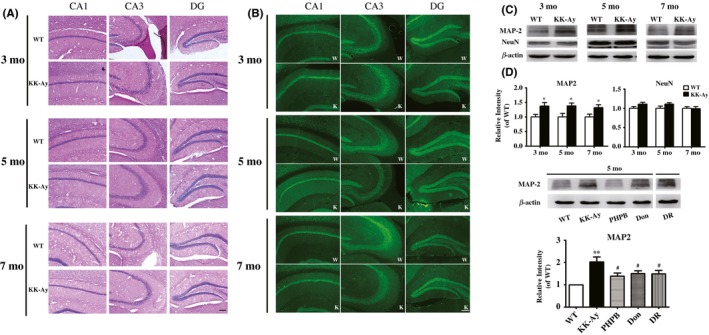
No loss of the number of neurons but increased expression of MAP2 in the hippocampus of KK‐Ay mice and the effects of PHPB, donepezil, and diet intervention (DR). A, Histological examination of HE staining of 3‐, 5‐, and 7‐mo‐old KK‐Ay mice. B, Representative image of neuron nuclei by immunofluorescence staining of NeuN in the hippocampus of mice of all ages (data of quantitative image analysis was not shown). C‐D, Representative image of western blotting of NeuN and MAP2 in the hippocampus of mice at different ages and quantitative analysis. E‐F, PHPB 150 mg/kg, donepezil (Don) 3 mg/kg, and DR decreased the expression of MAP2 in the hippocampus of 5‐mo‐old KK‐Ay mice (the lane of DR was in the same gel). Values were expressed as ratio to age‐matched WT (set to 1), n = 5‐8 mice per group. ^*^ *P* *<* .05, ^**^ *P* *<* .01 versus WT mice, ^#^ *P* *<* .05 versus KK‐Ay mice. Scale Bar = 100 μm

Hematoxylin‐eosin staining revealed that the neuronal nuclei of KK‐Ay mice organized closely and the cytoplasm were stained homogeneously (Figure 2A). No obvious abnormal nuclei and shrinkage in the size of both pyramidal cells (CA1 and CA3 region) and granule neuron (DG region) were seen in KK‐Ay mice. Together, there were no distinct abnormal changes in the distribution, morphology, and the number of the neurons in the hippocampus of KK‐Ay mice compared with WT mice. Neuronal nuclei (NeuN) as a specific nuclear protein in the mature neuron is an important marker of the neuron. In the present study, KK‐Ay mice at ages of 3‐7 month showed no significant change in the expression of NeuN compared to WT mice analyzed by both immunofluorescence staining and WB (Figure 2B‐D, data of quantitative immunostaining image were not shown). These results indicate that there was no observable change either in the number or in the morphology of hippocampal neurons in KK‐Ay mice.

### 3.3 Increased expression of MAP2 in the hippocampus of KK‐Ay mice and it was reversed by PHPB, donepezil, and diet intervention

The expression of microtubule‐associated protein 2 (MAP2), an important cytoskeleton‐associated protein in the neuron, was detected by WB (Figure 2C). In 3‐, 5‐, and 7‐month‐old KK‐Ay mice, the expression of MAP2 was significantly increased by 37.14%, 38.04%, and 31.06% (n = 5‐8, *P* *<* .05) in the hippocampus compared with age‐matched WT mice (Figure 2D). However, MAP2 was reduced in PHPB (150 mg/kg), donepezil (3 mg/kg), and DR groups of 5‐month‐old KK‐Ay mice (Figure 2E,F). It indicates that PHPB, donepezil, and DR could rescue the abnormality of MAP2.

### 3.4 Changes of astrocytes in the hippocampus of KK‐Ay mice

**Figure 3:**
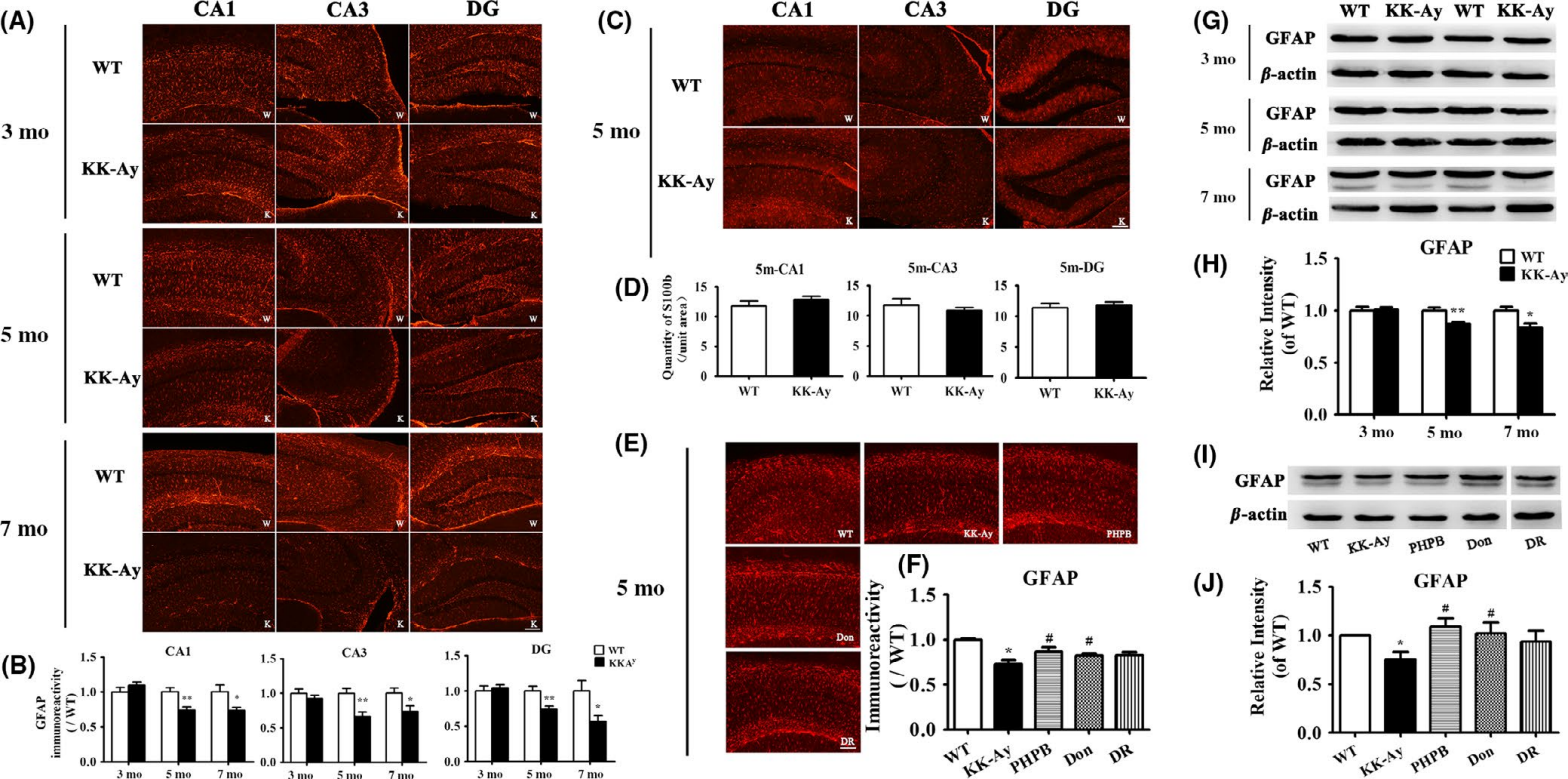
Abnormality in morphology and decreased expression of GFAP without a change in the relative number of astrocytes in the hippocampus of KK‐Ay mice and the effects of PHPB, donepezil, and diet intervention (DR). A‐D, Representative image of astrocytes by immunofluorescence staining of GFAP in the mice of all ages and S100b in 5‐mo‐old mice and corresponding quantitative image analysis. E‐F, PHPB 150 mg/kg and donepezil (Don) 3 mg/kg improved the morphological injury of astrocytes in the hippocampus of 5‐mo‐old KK‐Ay mice. G‐H, Representative image of GFAP by western blotting in the mice of all ages and quantitative image analysis. I‐J, PHPB and donepezil rescued the reduction of GFAP in 5‐mo‐old KK‐Ay mice (the lane of DR was in the same gel), n = 5‐8 mice per group. Values were expressed as ratio to age‐matched WT (set to 1). ^*^ *P* *<* .05, ^**^ *P* *<* .01 versus WT mice, ^#^ *P* *<* .05 versus KK‐Ay mice. Scale Bar = 100 μm

Glial fibrillary acidic protein (GFAP) is an astrocyte‐specific cytoskeleton protein which indicates the activation of astrocytes. As shown in GFAP immunostaining (Figure 3A,B), the GFAP‐positive astrocytes in the hippocampus of KK‐Ay mice at 5 and 7 months of age showed smaller cell body, shorter and thinner branches than age‐matched control mice. The expression of GFAP detected by WB also decreased in 5‐ and 7‐month‐old KK‐Ay mice (Figure 3G,H). But 3‐month‐old KK‐Ay mice presented no significant change both in the morphology of astrocyte and in the expression of GFAP. However, there were no significant differences in the relative number of hippocampal astrocytes between KK‐Ay and WT mice detected by the immunostaining of S100b (Figure 3C,D). It suggested that the decreased levels of GFAP in KK‐Ay mice are not due to the reduction in the number of astrocytes but resulted from the reduced size of astrocytes during the development of DE.

### 3.5 PHPB, donepezil, and diet intervention rescued the changes in GFAP expression and the morphology of astrocyte

After treatment with medicines or DR, KK‐Ay mice showed recovery in the morphology and GFAP expression in astrocytes detected by immunostaining and the expression of GFAP was higher in treatment groups than in KK‐Ay group (Figure 3E,F). PHPB 150 mg/kg and donepezil (Don) 3 mg/kg also obviously increased the expression of GFAP up to 44.72% and 35.75% versus KK‐Ay group detected by WB (Figure 3I,J, *P* *<* .05). These results revealed that treatment with PHPB or donepezil might protect the astrocyte against cytoskeleton damage.

### 3.6 Microglia was activated in KK‐Ay mice, and it was inhibited by PHPB, donepezil, and diet intervention

**Figure 4:**
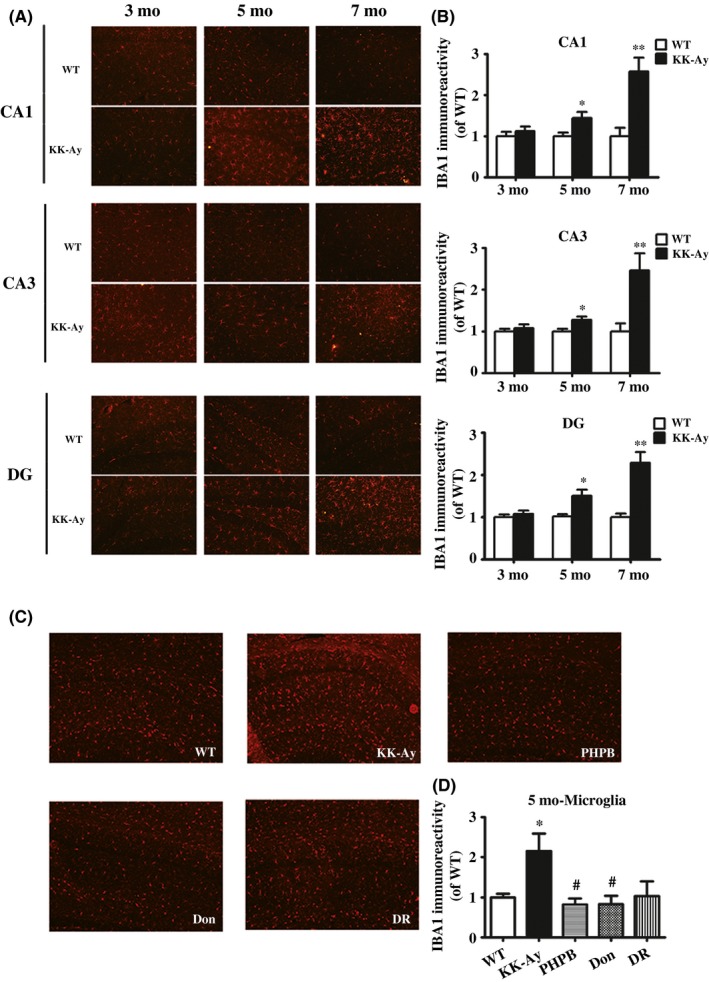
Activation of microglia in the hippocampus of KK‐Ay mice and the effects of PHPB, donepezil, and diet intervention (DR). A‐B, Representative image of microglia by immunofluorescence staining of IBA‐1 in the mice of all ages and quantitative image analysis. C‐D, PHPB 150 mg/kg, donepezil (Don) 3 mg/kg, and DR inhibited the activation of microglia in KK‐Ay mice, n = 5‐8 mice per group. Values were expressed as ratio to age‐matched WT (set to 1). ^*^ *P* *<* .05, ^**^ *P* *<* .01 versus WT mice, ^#^ *P* *<* .05 versus KK‐Ay mice. Scale Bar = 100 μm

Activated microglia is regarded as mainly neuroinflammatory cell type. We investigated the changes of microglia in the hippocampus by immunostaining of ionized calcium binding adapter molecule 1 (IBA‐1) which is a prominent marker indicating the activated microglia (Figure 4A,B). There was no significant change in the microglia in the hippocampus of 3‐month‐old KK‐Ay mice. However, higher IBA‐fluorescence intensity and the larger branches of cells showed in the hippocampus of 5‐month‐old KK‐Ay mice compared with age‐matched WT mice. At 7 months of age, the activation of microglia was more excessive in the hippocampus of KK‐Ay mice, with more amebic‐like cells. The data indicated that neuroinflammation enhanced in the hippocampus of KK‐Ay mice with the development of diabetes. Administration of PHPB 150 mg/kg or donepezil 3 mg/kg could significantly suppress the activation of microglia in 5‐month‐old KK‐Ay mice (Figure 4C,D). Diet intervention also showed a certain effect on the reduction of activated microglia.

### 3.7 Abnormal expression of transporters of glucose and glutamate in the hippocampus of KK‐Ay mice

**Figure 5:**
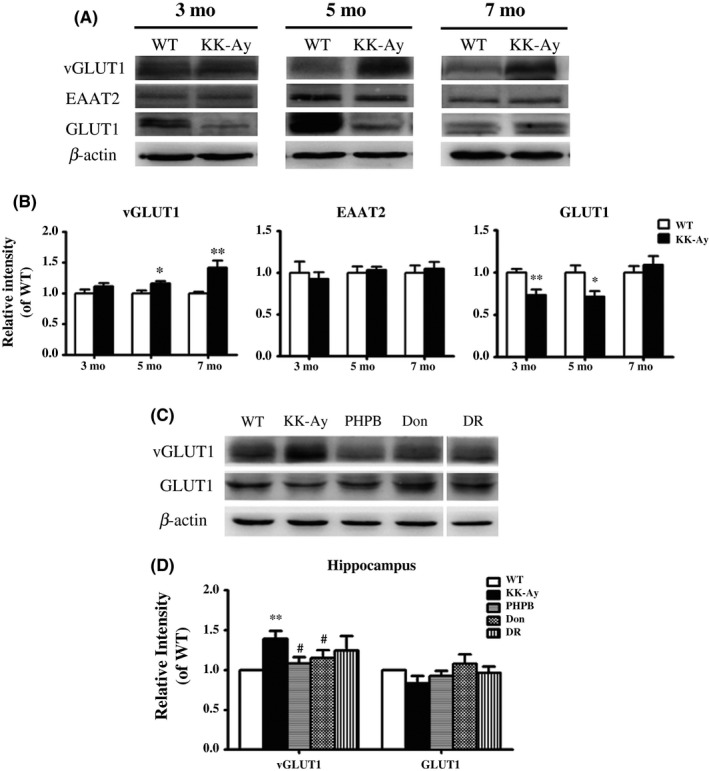
Effects of PHPB, donepezil, and diet intervention (DR) on the expression of GLUT1, vGLUT1, and EAAT2 in the hippocampus of KK‐Ay mice. A‐B, Representative image of GLUT1, vGLUT1, EAAT2 by western blotting in the mice of all ages and quantitative image analysis. C‐D, PHPB 150 mg/kg and donepezil (Don) 3 mg/kg rescued the abnormality of vGLUT1 in KK‐Ay mice but have no obvious effect on the GLUT1 (the lane of DR was in the same gel), n = 6‐9 mice per group. Values were expressed as ratio to age‐matched WT (set to 1). ^*^ *P* *<* .05, ^**^ *P* *<* .01 versus WT mice, ^#^ *P* *<* .05 versus KK‐Ay mice

Glucose transporter 1 (GLUT1, 45 kDa) is mainly expressed in the astrocyte and is mainly responsible for maintaining the stability of glucose metabolism. Compared with age‐matched WT mice, the expression of GLUT1 was significantly reduced by 26.42% and 28.49% in the hippocampus of 3‐ and 5‐month‐old KK‐Ay mice (Figure 5A,B). However, there was no obvious reduction of GLUT1 in KK‐Ay mice at 7 months of age. The data indicated that the GLUT1 might dynamically change in KK‐Ay mice with the metabolism disorder. In addition, the expression of vesicular glutamate transporter (vGLUT1) that specifically expressed in the presynaptic membrane to regulate the release of glutamate was obviously increased in the hippocampus of 5‐ and 7‐month‐old KK‐Ay mice up to 16.1% and 42.0%, but no obvious change in KK‐Ay mice at 3 months (Figure 5A,B). Another glutamate transporter referred to excitatory amino acid transporter 2 (EAAT2), which mainly expressed in astrocytes to take charge of eliminating redundant glutamate in the synaptic cleft, had no change in KK‐Ay mice compared to WT mice (Figure 5A,B). These results illustrate that both GLUT1 and vGLUT1 might involve in the pathology of DE in KK‐Ay mice.

### 3.8 PHPB and donepezil and diet intervention rescued the abnormality of vGLUT1 in KK‐Ay mice but have no obvious effect on the GLUT1

PHPB, donepezil, or diet changing appeared to reduce the expression of vGLUT1 in the hippocampus compared with the model group (Figure 5C,D). Particularly, PHPB 150 mg/kg and donepezil 3 mg/kg showed a significant decrease of vGLU1 in 5‐month‐old KK‐Ay mice by 22.0% and 17.3%, respectively. Although there were no significant differences in the expression of GLUT1 between treatment and KK‐Ay group, it could not exclude the effect of PHPB, donepezil, and DR on GLUT1 since the expression of GLUT1 in treated groups showed an elevation and was closed to the WT group.

### 3.9 Reductions of BDNF and GDNF and increase of IL‐1β and TNF‐α in the hippocampus of KK‐Ay mice and the recoverable effect of PHPB and donepezil and diet intervention

**Figure 6:**
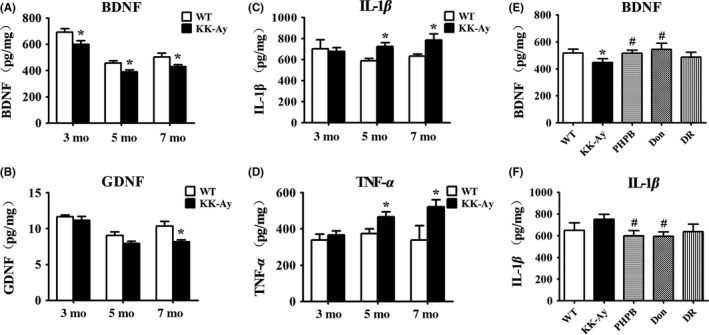
Reductions of BDNF and GDNF and increases of IL‐1β and TNF‐α in the hippocampus of KK‐Ay mice and the recoverable effects of PHPB, donepezil, and diet intervention (DR). A‐D, The level of BDNF, GDNF, IL‐1β, and TNF‐α detected by ELISA in the hippocampal homogenate of 3‐, 5‐, and 7‐mo‐old WT and KK‐Ay mice (n = 4‐6 mice per group). E‐F, The effects of PHPB 150 mg/kg, donepezil (Don) 3 mg/kg and DR on the abnormalities of BDNA and IL‐1β in the hippocampus of KK‐Ay mice (n = 6‐9 mice per group). Values were referred as the relative amount per total protein (pg/mg). ^*^ *P* *<* .05 versus WT mice, ^#^ *P* *<* .05 versus KK‐Ay mice

The level of BDNF in the hippocampus of KK‐Ay mice decreased at 3, 5, and 7 months of age. Similarly, the level of GDNF also showed a significant decrease at age of 7 months and the clear decline trend in 3 and 5 months age compared with WT (Figure 6A,B). On the contrary, the inflammatory factors IL‐1β and TNF‐α were significantly increased in the hippocampus of 5‐ and 7‐month‐old KK‐Ay mice (Figure 6C,D). Obviously, PHPB and donepezil could increase the concentration of BDNF and decrease the level of IL‐1β of KK‐Ay mice (Figure 6E,F). Diet intervention could also reduce the level of IL‐1β to some content.

## 4 DISCUSSION

Our study demonstrated that KK‐Ay mouse is a stable and useful model of T2DM and the mice showed obviously cognitive deficiency in Morris water maze test. It was similar to the previous studies, which KK‐Ay mice displayed a cognitive dysfunction at quite young with low activity of long‐term potentiation (LTP).^16^, ^21^, ^22^ However, diabetes‐induced dementia is different from AD in pathogenesis. The latter is mainly induced by neuronal degeneration. The metabolic disorder may often cause obvious changes in blood vessels and induce VD. Therefore, diabetes‐related dementia, also called DE, appears both VD‐ and AD‐like characteristics. In this study, we focused mainly on the morphological changes of the neurons and astrocytes, the expressions of glucose and glutamate transporters in neurons and astrocytes as well as the intervention of diet and medicines on DE.

Although some previous reports showed a significant loss of neurons in diabetic patients and in animal models,^2^, ^9^, ^12^, ^23^ we did not see obvious changes in morphology and number of neurons in the hippocampus of KK‐Ay mice in the present study. The difference between animal models might result from different species and the severity of the disease. Most DE studies used STZ‐induced diabetic rats, and the neuron loss might not completely reflect the effects of diabetes, since the direct neurotoxicity of STZ was confirmed by intracerebral‐ventricular injection.^24^, ^25^ In addition, treatment with STZ mainly damages pancreas and impedes the synthesis and secretion of insulin, which is distinguished from KK‐Ay mice with a high level of insulin resistance.

In the present study, we observed an increased expression of MAP2, an important cytoskeleton‐associated protein expressed in dendrite and perikarya of the neuron.^26^, ^27^ It is similar to the reports in AD patients 28 and in old mice with cognitive decline.^29^ However, it is different from some studies in DE, VD, and AD, which showed decreased MAP2 in the brain.^30^, ^31^ Some previous studies suggested that the reduction of MAP2 was coincident with neuron loss in the brain, so the decrease in MAP2 might result from the reduction of the number of neurons directly. It indicated that there was no obvious loss of neurons in KK‐Ay mice in our study. NeuN, the protein marker for neurons, was not changed from 3 to 7 month of age in our study. It also demonstrated that the number of neurons was not reduced. The mechanism of MAP2 upregulation here was still not clear.

Unlike the neurons in KK‐Ay mice, the astrocytes were changed at the early stage of diabetes. They exhibited a smaller size with the decreased expression of GFAP in the hippocampus of KK‐Ay mice. Similar results were seen in several previous studies of both T1DM and T2DM animal models.^32^, ^33^ Since GFAP^−/−^ mice appeal to impairment of LTP, imbalance of glutamate‐glutamine cycle, abnormal BBB, and demyelization, it has confirmed the importance of GFAP for the normal function of astrocytes that maintain and protect neurons.^34^, ^35^, ^36^ Therefore, the decrease of GFAP in KK‐Ay mice might play a role in cognitive dysfunction. Moreover, the level of BDNF and GDNF, considerably synthesized and secreted mainly by astrocytes, was significantly decreased in the hippocampus of KK‐Ay mice, with similar change trend of GFAP. The lower levels of BDNF/GDNF make cells more vulnerable under injury. Together, astrocytes might damage at the early stage of diabetes in KK‐Ay mice and it could be an important mechanism of DE.

In addition, neuroinflammation in CNS is also important in the development of AD, VD, and DE.^37^ The gradual activation of microglia was observed in the hippocampus of KK‐Ay mice with age. Although active microglia is actually beneficial in certain circumstances to stimulate cell repairing, remove toxic proteins and debris from CNS, continuous activation of microglia might induce damage of normal tissues and injury of CNS by releasing proinflammatory factors, such as IL‐1β, TNF‐α, and so on.^38^ In consequence, the concentrations of IL‐1β and TNF‐α increased in KK‐Ay mice, indicating chronic and progressive inflammation in the central nerves system would also play a role in DE of KK‐Ay mice.

The pathophysiology of the diabetes encephalopathy may closely relate to the glucose and glutamate transportation and utilization.^39^, ^40^, ^41^ The function of GLUT1 (45 kDa) is transport glucose, and the protein is mainly expressed in astrocytes. In the present study, the expression of GLUT1 was found attenuating in the hippocampus at 3‐ and 5‐ month‐old KK‐Ay mice, implying reduced glucose uptake from blood to astrocyte. This situation may cause a decrease of glucose utilization in astrocytes and subsequently reduce supports to neurons. Most of the energy and nutrition required by neurons obtained from astrocytes, and it was consistent with a change in the brain of AD, chronic hyperglycemia, and high‐fat dietary,^42^, ^43^ which GLUT1 is also down‐regulated. But the mechanism of down‐regulation of GLUT1 remains to be studied. Anyway, reduced expression and activity of GLUT1 on astrocytes might inhibit glucose transport and energy production; further, neurons become apoptosis or degeneration due to the insufficient supply. This would be related to the pathogenesis of DE or AD.

In the hippocampal neurons, glutamate is stored and released by vGLUT1, and then, it is ingested into astrocytes by EAAT2. A significant increase in vGLUT1 was observed in the hippocampus of KK‐Ay mice. Similar results were observed previously in primary cultured neuron and mice with high glucose or fat treatment in vitro and in vivo.^44^, ^45^, ^46^ Since increased vGLUT1 is linked to excess glutamate release,^46^ indicating a high risk of glutamate excitotoxicity in KK‐Ay mice. However, the expression of EAAT2 in the hippocampus showed no obvious change in KK‐Ay mice, implying the damage of neurons by glutamate mainly due to the glutamate store and release, but not due to the clearance.

Our study demonstrated that DE might be ameliorated by treatment of medicines such as donepezil and PHPB, as well as by DR (from high energy diet to normal diet). Donepezil, an acetylcholinesterase (AChE) inhibitor, has been proven to be beneficial to AD, ischemic injury, and VD. Except for its main pharmacological effects, donepezil could increase cerebral blood, inhibit neuroinflammation, increase neurotrophic factors, and reduce oxidative stress in the brain.^47^ Similar with donepezil, *dl*‐PHPB, a prodrug of *dl*‐NBP for ischemic stroke, could attenuate the cognitive impairment. Our previous studies have shown that *dl*‐PHPB significantly improves cognitive function in various animal models of dementia.^18^, ^19^, ^48^ The mechanisms of PHPB or *dl*‐NBP to treat neuronal degenerative diseases are involved in signaling pathways of PI3K/AKT, pCREB, and BDNF synthesis and release. Therefore, PHPB/NBP might also promote neurogenesis. 49 In addition, in the present study, we found that both donepezil and PHPB could attenuate the elevation of MAP2 and VGLUT1, rescued the reduction of GFAP, and increased neurotrophic factor in KK‐Ay mice. Meanwhile, both of them inhibited the activation of microglia with decreasing the proinflammatory factor.

Moreover, we also demonstrated that the cognitive deficits in KK‐Ay mice could be relieved by a low‐fat diet. It could be resulted by the recovery of blood glucose and fatty acids and cholesterol levels, subsequently relieved pathological changes in blood vessels. Our data indicate that the cognitive deficits of T2DM could be recovered at the early stage of the disease. It is different from AD that is almost irreversible. Donepezil and dl‐PHPB would be the potential therapeutic option for DE.

## 5 CONCLUSION

Diabetic encephalopathy study with KK‐Ay mice showed an early stage change in neurons and glial cells. Especially, the morphology of astrocytes and its structural marker protein GFAP changed significantly. Meanwhile, neuronal glutamate transporter vGLUT1 increased and astrocyte glucose transporter GLUT1 decreased. All the above pathological changes could be rescued by DR and by neuronal protective agent treatment. The alteration in blood vessels in DE remains to be studied in the future.

## CONFLICT OF INTEREST

The authors declare no conflict of interest.
